# Baseline Activity Patterns of Two Captive Red Pandas (*A. f. styani*) to Inform Future Conservation Translocations in Sichuan China

**DOI:** 10.3390/ani16111736

**Published:** 2026-06-04

**Authors:** Xueyang Fan, Ruiqing Lv, James Edward Ayala

**Affiliations:** Chengdu Research Base of Giant Panda Breeding and the Conservation of Endangered Wildlife Key Laboratory of Sichuan Province, 1375 Panda Road, Northern Suburb, Chengdu 610081, China

**Keywords:** red panda, activity patterns, translocation, ODBA

## Abstract

The endangered red panda (*Ailurus fulgens*) is endemic to the southeastern slopes of the Himalayas. Following the successful translocations of captive-born giant pandas in China, plans have begun to release captive-born red pandas into their habitat where the two species coincide. In order to provide baseline activity data, as part of pre-release preparation, a pair of captive-born red pandas were trained to allow the placement of GPS collars mounted with triaxial accelerometers. Ten months of Overall Dynamic Body Acceleration data were collected from the accelerometers, which showed that the pandas were primarily diurnal and had an activity rate of 42.32% (95% CI: 41.67–42.97), similar to that of wild red pandas. The animals in this study differed from wild individuals in having a distinct tri-modal diel pattern related to the captive feeding schedule. Changes in this pattern may serve as an indicator of whether a captive individual is suitable for release, and post-release resumption of the bi-modal pattern seen in wild populations may serve as an indicator of an individual’s adaptation to the wild. Though limited by the sample size, this study provides information for the management and preparation of captive red pandas for future translocations.

## Simple Summary

In order to collect baseline activity data in anticipation of future red panda translocations in China, a breeding pair of captive-born red pandas was trained using positive reinforcement conditioning to wear GPS collars mounted with triaxial accelerometers. Activity data in the form of Overall Dynamic Body Acceleration was collected and analyzed over ten months, which showed a similar activity rate to wild red pandas. A key difference was noted in daily activity patterns, with the captive pair of red pandas showing a tri-modal diel pattern compared to the bi-modal pattern of wild individuals. Changes in this pattern may serve as a useful pre-release indicator in deciding if an individual is suitable for translocation; in addition, resumption of this pattern post-release may show an individual’s successful adaptation to the wild.

## 1. Introduction

The red panda (*Ailurus fulgens*), a small arboreal carnivore, is endemic to the southeastern slopes of the Himalayas, across five range countries: India, Nepal, Bhutan, Myanmar, and China [1]. Red pandas are monophyletic and the only existent member of the family Ailuridae [2]. Two subspecies, *A. f. fulgens* and *A. f. styani*, are currently recognized by the International Union for the Conservation of Nature (IUCN); however, recent research suggests these may actually be two distinct species [3]. Within China, *A. f. fulgens* is only found in southern Tibet, whereas *A. f. styani* is found in southeastern Tibet, Sichuan, and Yunnan provinces [3]. While unrelated, red pandas and giant pandas (*Ailuropoda melanoleuca*) are both bamboo specialists that share virtually the same mountainous bamboo forests within Sichuan province [4].

As of 2026, the red panda is listed by the IUCN as “Endangered”, with the global population believed to be less than 10,000 individuals and declining [1]. Habitat loss/fragmentation and poaching are major threats to this species [5,6,7]. In China the red panda is considered a Class II conservation species. Wei et al. [8] reported during a survey between 1994 and 1996 that the estimated population of red pandas in China was between 6000 and 7000 individuals across the three provinces. However, due to the elusive nature of the species and the remoteness of its high-altitude montane habitat, a more recent population census is yet to be completed. In contrast, China has made great strides in the conservation of the giant panda, resulting in the downgrade of the species from “Endangered” to “Vulnerable”. The establishment of the 27,134 km^2^ Giant Panda National Park [9] was designed with the hope that giant pandas will serve as an umbrella species across its range [10]. Given the lack of contemporary red panda census data, the extent to which the red panda in Sichuan is benefitting from conservation efforts focused on habitats it shares with giant pandas is unknown.

Translocations of captive-born individuals have been a key component in the successful conservation of the giant panda [11,12]. As of 2021, 12 captive individuals have been released into the wild as a means to reinforce the small genetically isolated populations of wild giant pandas [12]. Given the success of captive-born giant panda translocations, coupled with the establishment of the aforementioned Giant Panda National Park, as well as increased environmental conservation legislation throughout the nation, plans have begun for the translocation of captive-born red pandas within China. To date, globally, there has only been one documented translocation of captive-born red pandas into the wild, which entailed the 2003 release of two females in India [13].

Activity patterns are an important aspect of animal behavior, and understanding activity patterns is vital in planning conservation strategies for a species, especially conservation translocations [14,15]. Activity patterns are related to several complex biotic and abiotic factors ranging from light conditions and ambient temperature, to interspecific competition, breeding seasons, predation, and food availability [16]. How quickly an animal transitions to a stable activity pattern post-translocation, and whether or not a captive animal is able to adjust its activity pattern to that seen in wild populations are useful indicators in post-release monitoring [17,18].

To document the activity patterns of wild red pandas, only three known studies were conducted using radio collars [19] fitted with motion-sensitive mercury switches. Each of the three studies used similar recording methods to detect and monitor activity data of *A. f. styani* in nature reserves within Sichuan, China [20]. First, Johnson et al. (1988) [4] monitored one young female red panda for nine months and reported primarily nocturnal activity, with some crepuscular activity and a small amount of diurnal activity. The red panda was reported active for 36.5% of the time. Later, Reid et al. (1991) [21] tracked an adult male and adult female red panda for nine months as well, and conversely reported that the pair of red pandas were more active during the day, followed by a moderate amount of crepuscular activity, and least active at night. A sex-related difference was noted, with the female reported to be active 49% of the day while the male was active 45% of the day. Lastly, Zhang et al. (2000) [20] used a larger sample size of six adult red pandas (three male, three female) across one full year and found a similar activity pattern (i.e., diurnal, crepuscular, nocturnal) to Reid et al. (1991) [21]. Conversely, the females were active 47.9% of the day, while the males were active 49.5% of the day.

The integration of triaxial accelerometers as a means to remotely measure wildlife activity became available for research in the late 1990s, and these early studies were conducted with captive and domestic animals, as well as on aquatic species in which it was difficult to conduct direct observations [22]. Unlike the older mercury switches, which can only record whether an animal is active or inactive, tri-axial accelerometers simultaneously measure an animal’s rate of acceleration in space along three dimensions, X, Y, and Z, which then can be measured and formulated into Overall Dynamic Body Acceleration (ODBA) to identify several different levels of active states displayed by an animal [15,23,24]. Because of the ability to accurately record different activity states, GPS collars equipped with accelerometers have become the current standard for remotely recording activity from wildlife and have been used with approximately 125 species [22].

Therefore, the objective of this study was to collect the baseline activity data of captive red pandas in a naturally forested enclosure within the species’ native habitat range in preparation for future translocations. To do this, a breeding pair of red pandas was selected from the captive population at the Dujiangyan Field Research Center for Giant Pandas (Panda Valley) in Sichuan, China. These individuals were housed in an isolated nonpublic access breeding enclosure and trained using positive reinforcement for the voluntary placement of GPS collars fitted with triaxial accelerometers without any chemical immobilization or physical restraint. This is the first study of its kind to collect and analyze activity data from captive red pandas using accelerometers, and provides information for the captive management of the species as well as insights into the preparation of captive red pandas for future translocations.

## 2. Materials and Methods

### 2.1. Study Area

Data were collected between April 2025 and January 2026 at Panda Valley, Dujiangyan, 30.9677056° N, 103.5785984° E. Panda Valley is approximately 20 km from the Longxi-Hongkou National Nature Reserve, and it is located in the foothills of the Qionglai mountains, part of both the core red panda, and giant panda habitat in Sichuan province. This facility was built as a reintroduction, pre-release acclimation, and research facility specifically for giant pandas. However, in 2017, a large 1.5 hectare (15,000 m^2^) red panda enclosure was built and opened to the public in 2019. The entire area was constructed on the slope of a mountain and is completely naturally forested with large native tree species. This area is open to the public throughout the year from 8:00 to 17:30 daily. The two red pandas in this study were housed in a separate 100 m^2^ fenced-in isolated breeding/maternity enclosure. This isolated enclosure was built higher up the slope of the main enclosure. Due to the slope, the height in the enclosure was irregular, with the highest portion of the fence being 3.8 m, and the lowest portion being 2 m high. Three medium sized native trees (all *Magnolia officinalis*) were allowed to grow out of the top portion of the enclosure to provide shade; additionally, several smaller trees (*Magnolia officinalis* and *Phoebe zhennan*) along with several native shrub species were present. Three nest boxes, as well as added perching and climbing structures were installed to increase vertical complexity and support the nest boxes. This yard is attached to the main public access area with a buffer zone of 44 m of forest between it and the nearest public walkway to reduce disturbance.

Throughout the study period, the two red pandas were provisioned with fresh bamboo four times a day (8:00, 10:00, 13:30, and 16:30). *Bashania fargesii*, *Phyllostachys bissetii*, and *Chimonobambusa opienensis* are the three most common species of bamboo provided, with *Chimonobambusa pachystachys*, *Chimonobambusa purpurea*, *Indocalamus tessellatus*, and *Pleioblastus amarus* provided depending on availability. In an effort to match the seasonal diet of wild red pandas, the two captive red pandas were fed the stems with leaves and shoots of these species as they occur naturally throughout the year. In addition to bamboo, each red panda was provisioned twice a day with a dough-like mixture made from wheat, oats, corn, soybeans, rice, vegetable oil, and essential minerals and vitamins as a nutritional supplement. Fresh produce such as apples and pumpkins was provided as part of the normal diet as well as for environmental enrichment and positive reinforcement training. Water was provided via water bowls in the study area. Brief cleaning of the enclosure occurred simultaneously during each feeding session.

### 2.2. Subjects

A breeding pair of adult red pandas (*A. f. styani*) was selected from the captive population at Panda Valley. These individuals were selected based on their initial behavior (i.e., climbing ability, reaction to novel staff members), age, health, and genetic compatibility. Age selection was based on data from the red panda reintroduction in India in which a five-year-old and six-year-old female were released [13]. The male red panda in this study was born in 2020 and was four years old at the time the study began, and the female was born in 2021 and was three years old at the beginning of the study. Both pandas were mother-reared, and the female birthed a litter of twins the summer of 2024, which she weaned prior to beginning the study. Both red pandas were healthy and did not receive medication or treatment during the study period.

### 2.3. GPS Collars

Lightweight Druid (Druid technology Co., Ltd., Chengdu, China) Interrex B5 4G MC custom-made GPS collars were used for this investigation. The collars weighed 84.5 g and used the 4G cellular network. The red pandas in this study weighed ~6 kg, therefore the 84.5 g collars were 1.4% of the red panda’s bodyweight, considerably lower than the 5–10% accepted range commonly used in wildlife research [25].

To ensure ample battery power and check the safety of the red pandas, collars were changed on a weekly basis. The collars were set to record ODBA data at 1 min intervals for 24 continuous hours over the course of the week. Two collars were used per red panda, so that one collar would charge while the other was worn by the panda. To remove any effect of the collar change process on the activity data of the red pandas the first 15 min of the data from the new collar was deleted as was the last 15 min of the data from the older collar.

### 2.4. Training for GPS Collar Placement

Van De Bunte et al. [26] noted a significant change in behavior related to the placement of GPS collars on captive red pandas at Rotterdam Zoo, The Netherlands; therefore, to compensate for this, lightweight collars were selected, and a detailed plan was made to use positive reinforcement training for each panda to gradually desensitize and habituate the placement of the GPS collars without anesthesia or any physical restraint. At no point during the study period were the animals captured, netted, trapped, or physically held; participation was entirely voluntary, and an animal was free to decline participation or leave during any session. In brief, to place the GPS collars, the red pandas were first trained to stay in a fixed location (station) and then reinforced to hold this position. While in this fixed location, a single trainer then desensitized the red panda by gently touching their heads and neck. Next, a secondary trainer would reinforce the red panda while the primary trainer placed the GPS collar and immediately removed it. For the next step, the collar was left on for ~1 min while other routine behaviors were trained with the red pandas, then immediately removed. Gradually, the period the red pandas wore the collar was extended, until the collars could be left on the red pandas for 24 h.

The red pandas were initially trained twice a day at 10:30 and 14:00 three times a week. Each step took several days, and the entire process took approximately two months. No alternations were made to the normal red panda diet or feeding schedule for training purposes. Apple slices and tactile reinforcement were used as primary reinforcers; a training whistle was used as a bridge (secondary reinforcer). To ensure safety during the habituation process to the GPS collars the red pandas were continuously monitored by staff throughout the entire habituation period. Once habituated, the collars could be changed by a single trainer in under one minute.

Post habituation, the training was reduced to one session two to three times a week at 10:30 to maintain behaviors, check the collars to ensure proper fit and change the collars based on battery level. A small but acceptable amount of fur was abraded from the dorsal surface of the red pandas’ neck; however, no broken or irritated skin was noted during the entire study period. No abnormal behaviors or collar-directed behaviors were noted during this study following habituation. All data collection occurred after the animals were habituated to the collars.

### 2.5. Statistical Analysis

#### 2.5.1. Data Preparation and Analytical Unit

Raw collar output was sampled at 1 min intervals as integer ODBA counts; we converted these to ODBA in units of g (gravitational acceleration) by dividing by 10,000. Because ODBA integrates acceleration arising from all body movement, it indexes overall movement intensity rather than locomotion per se; the “active” classification used throughout this study therefore reflects any non-rest movement—including small-amplitude movements made while stationary—rather than locomotion in the strict sense. Records with missing or non-positive values were excluded. All timestamps were handled in China Standard Time (UTC +08:00).

The collars recorded continuous minute-level ODBA from 7 April 2025 to 12 January 2026 for both individuals (281 calendar days). After exclusion of invalid minutes, due either to equipment failure or human error, 707,258 min-level records (~11,788 h) entered the analysis: 252 days for the male and 255 days for the female. No data was excluded due to meteorological events. Meteorological variables were not recorded or modeled as covariates by design: this study aimed to characterize baseline activity relative to the captive feeding and management schedule rather than to identify the environmental drivers of activity, although the principal abiotic driver of activity rhythm and photoperiod was examined separately (Section 2.5.5). Because the open-topped enclosure exposed the animals to ambient conditions within the natural climatic range of the subspecies, weather varied naturally across the study period; isolating the contribution of individual meteorological variables would require their explicit measurement and is identified as a direction for future work (Section 4).

Minute-level records within the same hour are strongly autocorrelated and cannot be treated as independent observations. To avoid pseudoreplication, all inferential models used the hour as the analytical unit, with the response variable encoded as the pair (active minutes, inactive minutes) within each hour. Hours containing fewer than 30 valid minutes (i.e., <50% data coverage within the hour) were excluded, leaving 11,802 hourly bins. The 1 h window was chosen because it is short enough to resolve the diurnal rhythm at the temporal resolution of the four-times-daily feeding schedule, and long enough to render successive units approximately independent given the empirical bout-duration distribution (Figure S1, Table S1).

#### 2.5.2. Activity-Intensity Classification by Hidden Markov Model

**Figure 1:**
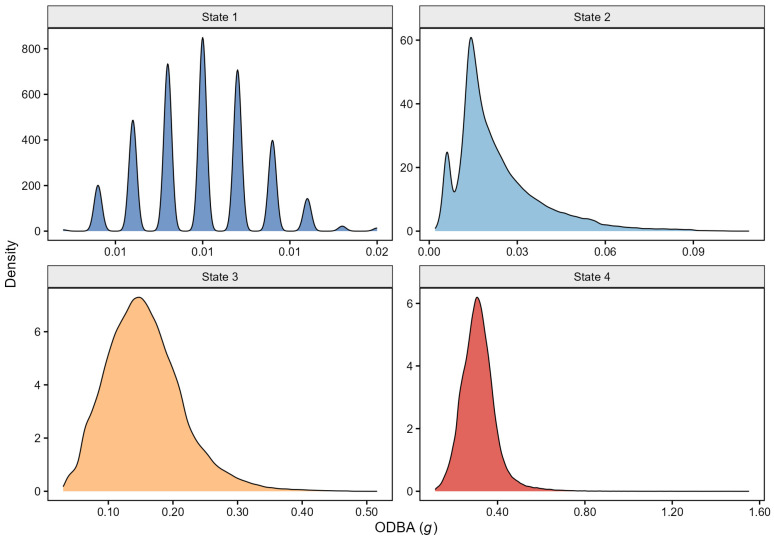
Distribution of ODBA values among the four activity-intensity states identified by the Hidden Markov Model. Each panel shows the density distribution for one state with independent y-axis scaling. State 1 corresponds to the lowest movement intensity and State 4 to the highest. The empirical correspondence between states and observable behaviors is provided in Section 3.2.

We classified each minute of ODBA into a discrete activity-intensity state using a Hidden Markov Model (HMM) implemented in the momentuHMM R package [27]. State-conditional ODBA was modeled with a Gamma emission distribution; this choice reflects the strictly positive support of ODBA and the well-known right skew of accelerometer signals, and it matches established practice for activity-state HMMs in mammals [28]. We fitted models with K = 1 to 5 states. To mitigate the risk of converging to local optima, each fit was repeated three times with random perturbations of the initial state-conditional means and standard deviations, and the fit minimizing the negative log-likelihood was retained. Models were ranked by AIC and BIC (Table 1). The four-state model achieved by far the lowest AIC and BIC and was selected as the optimal representation; the marked likelihood improvement at K = 4 is consistent with the four well-separated ODBA regimes visible in Figure 1.

The selected model was refitted on the full dataset (*n* = 707,258 min pooled across the two individuals). Hidden states were decoded using the Viterbi algorithm and renumbered in ascending order of state-conditional mean ODBA, so that State 1 corresponds to the lowest movement intensity and State 4 to the highest. We refer to them throughout as activity-intensity states. Goodness of fit, assessed using normal pseudo-residuals, was adequate (Figure S3); the biological correspondence of the states was assessed separately by direct video observation, as described next.

#### 2.5.3. Behavioral Validation of HMM Activity-Intensity States

To assess the biological correspondence of the four HMM states, focal continuous video observations were conducted on 8 January 2026 between 09:00 and 17:00 (China Standard Time), spanning diurnal and crepuscular hours. Both individuals were observed (male: 293 min; female: 88 min; total 381 min), all within the period of active collar deployment, so that each observed minute could be linked to a Viterbi-decoded HMM state and the corresponding ODBA value. Observations were made by a single observer through existing facility cameras to avoid observer presence in the enclosure. Each minute was divided into four 15 s intervals, and at each 15 s checkpoint the observed behavior was recorded using a pre-defined ethogram with eight codes: r (rest, lying flat with no visual movement); sa (stationary alert—sitting, lying or standing with only small head, ear, or tail movements); g (groom); scr (scratch head with hind legs); e (eat); w (slow stop-and-start exploratory walking); f (fast walking); c (climb, walk on tree branches, or jump on/off branches). Two further codes (os, oa) were used when the animal was out of sight (behind a tree or in the box). Codes denoting the same action with minor spelling variants (ea → e) were merged.

Each of the eight observable behaviors was mapped a priori to one of four intensity layers chosen to match the four HMM states by ODBA magnitude rather than by behavioral meaning: Still (r), Low (sa), Medium (g, scr, e, w), and High (f, c). The mapping was specified before any analysis of the joint distribution. Minutes for which two or more of the four 15 s labels were out of sight (os/oa) were excluded entirely (*n* = 15 min); for the remaining 366 min, individual 15 s labels of os/oa were dropped, yielding 1441 fifteen-second observations across the eight ethogram codes.

Independence between observed behavior (or intensity layer) and HMM state was tested using chi-square tests; when any expected cell count was below 5, the chi-square statistic was retained but the *p*-value was obtained by Monte Carlo simulation (B = 10,000 permutations under the null of independence [29]) rather than from the asymptotic chi-square distribution. Effect size was reported as bias-corrected Cramér’s V [30], which is preferred over the *p*-value because the four 15 s labels within a minute share a single HMM state and ODBA value and are not statistically independent; the reported *p*-values are therefore evidence against complete independence rather than calibrated significance tests, and Cramér’s V is the primary inferential statistic. Agreement between the observed intensity layer and the layer implied by the HMM state (State 1 → Still, State 2 → Low, State 3 → Medium, State 4 → High) was summarized by Cohen’s κ [31] in unweighted form, by linear-weighted κ for ordinal data [32], and by exact-match accuracy and within-±1-layer accuracy. As a robustness check that removes within-minute autocorrelation entirely, the analysis was repeated at the minute level (*n* = 366), with each minute assigned the highest-intensity 15 s label observed within it.

#### 2.5.4. Activity Rhythms and Photoperiod

For all rhythm and rate analyses, each minute was binarized into active (HMM States 3 or 4) versus inactive (States 1 or 2). Civil-time photoperiod was assigned using local sunrise and sunset at the study site (30.67° N, 104.06° E) computed by the suncalc R package [33], with the morning crepuscular window defined as one hour before to two hours after sunrise, the evening crepuscular window as two hours before to one hour after sunset, and the diurnal and nocturnal windows as the remaining daytime and night-time periods, respectively.

Calendar season was assigned according to the convention used in previous wild red panda studies [20]: spring (April–June), summer-autumn (July–October), and winter (November–March). Hours were summarized into year-month bins for the seasonal-time-series analysis.

#### 2.5.5. Statistical Models

All inferential models were beta-binomial generalized linear mixed models (GLMMs) fitted in glmmTMB [34]. The response variable was the within-hour count of active minutes out of valid minutes; the beta-binomial family was used to accommodate the over-dispersion expected when many minutes are aggregated within an hour. Each model included a random intercept for date to absorb residual within-day correlation among the 24 hourly bins of a single calendar day. Marginal predicted activity probabilities and asymmetric 95% confidence intervals on the response scale were obtained by back-transforming model-based estimates through the logit link using the emmeans R package [35].

Four models were fitted, each addressing a different aspect of the activity profile:

(i) Diurnal rhythm—the binarized activity series was modeled as a function of hour of day (factor with 24 levels), to estimate hourly mean activity probability and produce an activity curve
(ii) Individual contrast—a model with a single fixed effect for individual identity (sex), used to estimate each animal’s mean predicted activity rate and the contrast between them.
(iii) Individual × season—a model with the interaction between individual identity and season, used both to test whether the contrast between the two animals varies across seasons and to derive seasonal predicted activity rates with 95% Cis.
(iv) Individual × month and individual × hour—the same structure with month or hour replacing season.

Because the design contains a single male and a single female, sex is structurally confounded with individual identity; we therefore did not interpret the fixed-effect coefficients labeled ‘sex’ as estimates of a population-level sex effect, but as the descriptive contrast between these two specific animals. The standard regression test statistics are reported for completeness in Section 3, but the primary basis for inference about the magnitude of the individual contrast is Cohen’s h, computed from the back-transformed predicted probabilities (h = 2·[arcsin(√p_1_) − arcsin(√p_2_)]) and interpreted using the conventional thresholds (|h| < 0.2 = negligible, |h| ≈ 0.2 = small, |h| ≈ 0.5 = medium [36]).

(v) Photoperiod—a model with photoperiod (three levels: diurnal, crepuscular, nocturnal) as a single fixed effect, fitted on the same hourly response variable with date as a random intercept. Predicted activity probabilities for the three windows, pairwise odds ratios (Tukey-adjusted) and pairwise Cohen’s h effect sizes are reported in Table S4 of the Supplementary Material.

#### 2.5.6. Anticipatory Activity Around Feeding Events

To examine whether the diurnal activity peaks were temporally entrained to the four daily feeding events (08:00, 10:00, 13:30, 16:30), we computed event-triggered averages of minute-level activity in a ±45 min window around each feeding time (Figure S2).

#### 2.5.7. Software

All analyses were performed in R version 4.4 [37]. The HMM was fitted with momentuHMM [38]; GLMMs with glmmTMB [34]; estimated marginal means with emmeans [35]; sunrise/sunset times with suncalc [33]; chi-square independence tests with Monte Carlo *p*-values via the base R chisq.test function [29]; and figures with ggplot2 [39].

## 3. Results

### 3.1. Activity-Intensity States

**Figure 2:**
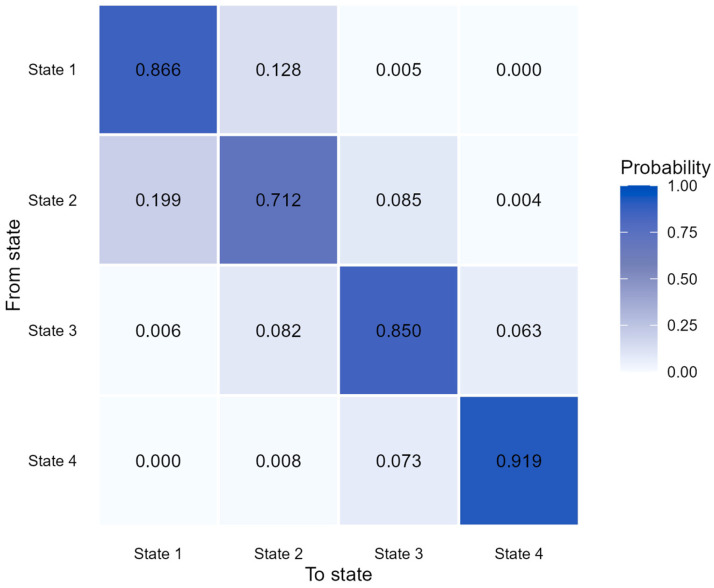
Transition probabilities among the four activity-intensity states estimated from the Hidden Markov Model. Darker colors indicate higher transition probabilities.

The four-state HMM was clearly preferred over models with one to three or five states by both AIC and BIC (Table 1). The state-conditional ODBA distributions were well separated and ordered by movement intensity (Figure 1): State 1 captured a tight peak of near-zero ODBA values; State 2 covered low-ODBA values; State 3 spanned an intermediate range; and State 4 captured the upper tail with substantially higher ODBA. Self-transition probabilities were high (Figure 2), with the strongest persistence in State 4 (0.919), indicating that activity intensity tends to remain stable from one minute to the next rather than alternating rapidly.

### 3.2. Behavioral Validation of HMM Activity-Intensity States

**Figure 3:**
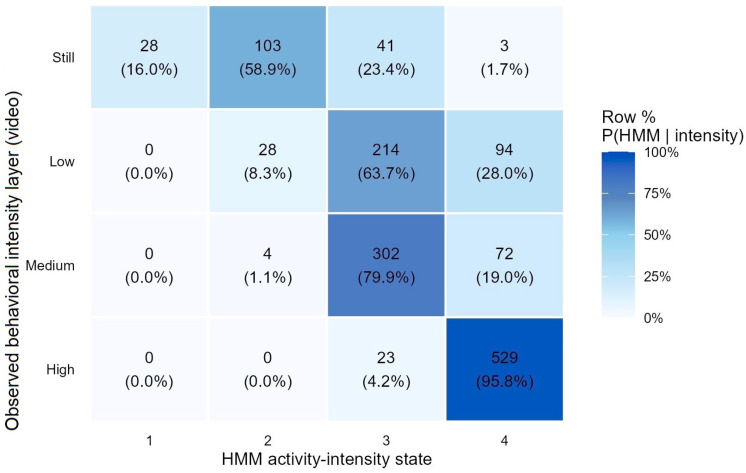
Behavioral validation of HMM activity-intensity states. Heatmap of *n* = 1441 fifteen-second observations, cross-classified by observed behavioral intensity (rows; pre-defined a priori from the ethogram codes) and Hidden Markov Model state (columns, numbered 1–4). Cells are colored by row percentage—P(HMM state observed intensity); cell labels show raw counts and row percentages. Association statistics are reported in Table 2 and Section 3.2.

Direct observation of behavior during a single 8 h validation session (8 January 2026; 366 min after exclusion of out-of-sight minutes; 1441 fifteen-second observations) confirmed that the four HMM states recover an ordered gradient of activity intensity. Observed behavioral intensity and HMM state were strongly associated (chi^2^ = 1555.65, Monte Carlo *p* < 0.001; Cramér’s V = 0.599; Table 2, Figure 3). The minute-level robustness analysis (*n* = 366) gave a comparable result (Cramér’s V = 0.679; linear-weighted κ = 0.733; exact-match accuracy = 77.0%), indicating that the association is not a statistical artifact of the within-minute autocorrelation discussed in Section 2.5.2.

The four states differed clearly in their behavioral composition (Figure 3, row percentages). State 4 corresponded to high-intensity activity in 95.8% of cases, almost entirely fast walking and climbing. State 1 contained no observations of medium- or high-intensity behavior; all 28 of its observations were of resting behavior. The intermediate states were less crisp: State 3 was a heterogeneous bin in which 52% of observations were of medium-intensity behavior (mostly grooming, eating, and slow exploratory walking), 37% were of stationary-alert behavior, and the remainder were of resting or high-intensity behavior, consistent with its position as an intermediate ODBA regime.

One feature of the table merits explicit comment. Behaviorally still observations (resting, lying flat) were more often classified as State 2 (58.9%) than as State 1 (16.0%). State 1 is therefore a strict subset of “complete immobility” rather than a general “rest” category—the HMM places most rest-like minutes into State 2, distinguishing State 1 only when ODBA is at its absolute minimum. Overall exact-match agreement between the observed intensity layer and the layer implied by the HMM state was 61.6% (Cohen’s κ = 0.438, moderate); when the intensity scale is treated as ordinal, 90.4% of 15 s observations fell within ±1 layer of the implied HMM intensity (linear-weighted κ = 0.527).

The active/inactive binary classification used in all subsequent analyses (Active = States 3 or 4) was strongly associated with locomotor versus stationary behavior (Cramér’s V = 0.751; Table S3). The classification, however, captures a broader notion than locomotion alone: stationary-alert behavior (small head, ear, and tail movements) was predominantly classified as Active, reflecting the sensitivity of ODBA to fine-scale movements [40]. The binary “Active” label as used in this study therefore corresponds to “any non-rest movement” rather than to “locomotion” in the strict sense.

These observations validate the HMM states as a graded measure of activity intensity that maps onto observable behavior with substantial-to-strong agreement, particularly at the high-intensity end (State 4 ↔ locomotion and climbing) and at the immobility end (State 1 ↔ deep rest). The intermediate states (State 2, State 3) capture mixtures of behaviors that share similar ODBA magnitudes but differ in their precise behavioral meaning; we therefore retain the activity-intensity framing of the states throughout the manuscript and avoid behavioral names. The validation was conducted on a single 8 h session and on two individuals; we expect, but cannot demonstrate from these data, that the boundaries of the correspondence will be similar in other seasons and individuals.

### 3.3. Diurnal Activity Pattern

**Figure 4:**
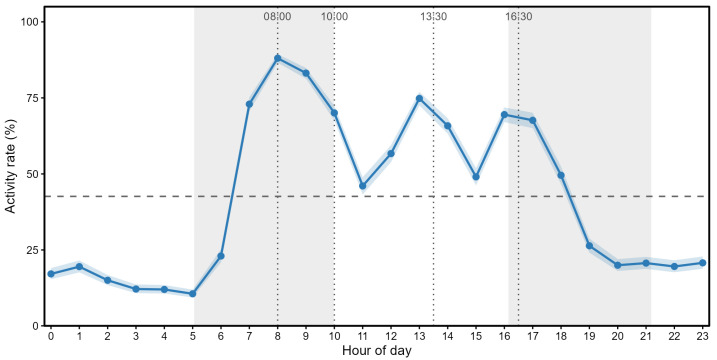
Mean diurnal activity pattern of the two red pandas studied. The blue line and shaded ribbon show the model-estimated hourly activity rate with 95% confidence intervals from a beta-binomial generalized linear mixed model. The horizontal dashed line represents the overall mean activity rate (42.32%). Vertical dotted lines mark the four daily feeding times (08:00, 10:00, 13:30, and 16:30). Light gray bands span the seasonal extremes of the morning and evening crepuscular windows.

The overall mean activity rate across the two individuals was 42.32% (95% CI: 41.67–42.97). The diurnal profile was tri-modal (Figure 4): a morning peak around 08:00–10:00, a midday peak around 13:00–14:00, and an afternoon peak around 16:00–17:00. The three peaks were tightly aligned with the four daily feeding events at 08:00, 10:00, 13:30, and 16:30, and event-triggered averages around each feeding time showed a clear anticipatory rise in activity in the minutes preceding each scheduled feed (Figure S2). Activity was highest during the diurnal and crepuscular hours and substantially lower at night.

**Figure 5:**
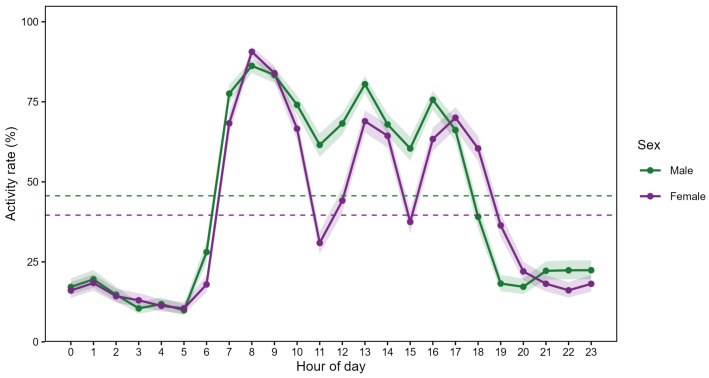
Activity probability for the two red pandas across the 24 h cycle. Lines show the model-estimated hourly activity rate for each individual with 95% confidence intervals (shaded ribbons) from a beta-binomial generalized linear mixed model with an individual × hour interaction. Horizontal dashed lines represent the overall mean activity rate for each individual.

Although the descriptive label “primarily diurnal” applies to the broad envelope, the precise location and amplitude of the three peaks coincide with the provisioning schedule, and the anticipatory rises (Figure S2) indicate that the animals had learned the timing of each feeding event. We therefore interpret the tri-modal pattern as reflecting entrainment of activity to the daily feeding schedule rather than an unconstrained endogenous rhythm. The two animals shared this tri-modal envelope but differed in baseline level, particularly during the night and early-morning hours (see Section 3.4. and Figure 5).

### 3.4. Contrast Between the Two Individuals

The model-estimated mean activity probability was 43.78% (95% CI: 42.87–44.70) for the male and 40.85% (95% CI: 39.95–41.76) for the female (Table 3). The corresponding Cohen’s h was 0.059, well below the conventional threshold for a small effect (|h| = 0.2). Across the 24 h cycle the two animals shared the same tri-modal envelope but the female showed deeper inter-peak troughs around 11:00 and 15:00, while the male maintained a slightly higher activity floor (Figure 5). Because the present study includes one male and one female, this contrast describes the difference between the two subjects during the study period and cannot be partitioned into a sex effect and an individual effect.

### 3.5. Seasonal Change in the Individual Contrast

When the individual × season interaction was modeled (Table 4), the contrast between the two animals varied across seasons. In spring, the female was very slightly more active than the male (Cohen’s h = −0.023, negligible); in summer-autumn the male was somewhat more active than the female (h = 0.082, small); and in winter the male was more active still (h = 0.125, small) (Table 5). The corresponding seasonal predicted activity rates were: spring, male 44.81% (43.20–46.43) and female 45.94% (44.36–47.54); summer-autumn, male 42.98% (41.53–44.45) and female 38.93% (37.48–40.40); winter, male 43.68% (42.01–45.38) and female 37.56% (35.96–39.19) (Table 5). All three within-season effect sizes fell below |h| = 0.2.

**Figure 6:**
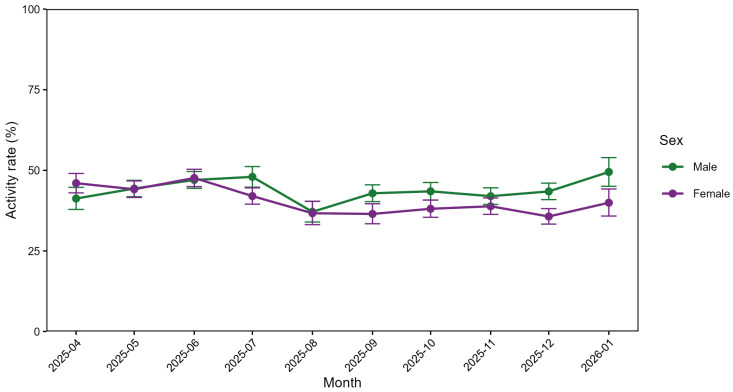
Monthly activity rate (%) for the two red pandas studied. Points and lines show model-estimated monthly activity probabilities; error bars represent asymmetric 95% confidence intervals from a beta-binomial generalized linear mixed model with an individual × month interaction.

The seasonal interaction terms were statistically significant in the regression sense (z = −3.35 and −4.41 for the summer-autumn and winter interactions, respectively; Table 4), reflecting the very large effective number of hourly observations rather than a large biological magnitude. The pattern across months (Figure 6) confirms this: the male maintained a relatively flat monthly trajectory across the ten-month sampling window, whereas the female declined progressively from ~46% in spring to ~38% in winter. With one male and one female we cannot resolve whether this divergence reflects a sex effect, an individual difference between the two subjects, or post-reproductive recovery in the female, who gave birth in summer 2024 and remained with her cubs into early 2025.

### 3.6. Photoperiod

Activity was concentrated in the daylight and twilight hours and was substantially reduced overnight. Based on the photoperiod GLMM (Section 2.5.5), the model-estimated mean activity probability was 63.61% (95% CI: 62.59–64.61) during diurnal hours (*n* = 4074 hourly bins), 60.75% (95% CI: 59.22–62.26) during crepuscular hours (*n* = 1920 bins), and 21.71% (95% CI: 21.01–22.42) during nocturnal hours (*n* = 5808 bins) (Table S4). Activity during the nocturnal period was substantially lower than during both the diurnal and crepuscular periods (Cohen’s h = 0.88 and 0.82, respectively; both large effects; Tukey-adjusted *p* < 0.0001 in each case), whereas the diurnal–crepuscular contrast was negligible (Cohen’s h = 0.06; Tukey-adjusted *p* = 0.004). The morning- and evening-crepuscular bands shifted across the year as the photoperiod lengthened and shortened (Figure 4), but the alignment of the activity peaks with the four feeding events remained the dominant feature of the diurnal profile in every season.

## 4. Discussion

Here the activity pattern of captive red pandas in a naturally forested enclosure is described using GPS collars mounted with triaxial accelerometers. Across ten months, over 11,800 h of ODBA activity data was collected. It was found that the daily activity rate for the two captive red pandas was 42.32% (95% CI: 41.67–42.97) (Figure 4), which is similar to the previously reported activity data from radio-collared red pandas in the wild within Sichuan province (36.5–49.5%) [20]. Typically, the activity of captive animals is lower than that of their wild counterparts. This lower activity level amongst captive animals is attributed to numerous factors such as limited space, lack of environmental complexity, reduced foraging/hunting time due to the provisioning of food, dissimilar climates as well as human interference among other factors [41,42]. While it is difficult to make a direct comparison due to differences in the environment and recording methods, a recent study by Bugler et al. [43] noted the 24 h activity level of captive red pandas (*A. f. fulgens*) in three zoos within Australasia to be 19–36%, lower than wild red pandas and the two captive individuals presented in this study. Bugler et al. [43] relied on focal observations during the day and video recordings during the night to describe activity patterns across three institutions. As a result, it is possible that activity was underreported due to behaviors being missed while the animals were out of view or during periods when the animals were not observed as opposed to the continuous, uninterrupted recording of a collar.

The lower activity level reported from these zoos in Australasia was partially attributed to decreased foraging time in captivity, as well as two of the seven subjects being geriatric (16 years old) and under anti-inflammatory medication, along with obvious differences in the environment [43]. Additionally, these zoo-housed red pandas were on public display, which likely further influenced their activity levels, whereas the animals in this current study were housed in an off-exhibit, naturally forested enclosure. Lastly, the animals observed by Bugler et al. [43] were *A. f. fulgens,* with the implication that there may be behavioral and/or anatomical differences between the two subspecies.

In regard to daily activity patterns, Zhang et al. [20] reported a bimodal activity pattern similar to Reid et al. [21], that peaked in the morning (07:00–10:00) and again at dusk (17:00–18:00), with the red pandas being primarily diurnal, with moderate crepuscular activity and least active at night. Johnson et al. [4] yielded different results, likely because the radio-collared individual was a sub-adult female that had not established a home range. While the two adult captive red pandas in this current study showed similar patterns to Zhang et al. [20] and Reid et al. [21] in regard to diurnal, crepuscular, and nocturnal activities, they differed from the wild red pandas and displayed a distinct tri-modal pattern with activity peaks at 08:00–10:00, 13:00–14:00, and 16:00–17:00 (Figure 4). This tri-modal pattern is likely due to the provisioning schedule of the red pandas at Panda Valley (Figure 4 and Figure S2). ODBA rose 30–45 min before the 08:00, 13:30, and 16:30 feedings, which can be interpreted as anticipatory behavior. This likely indicates that both red pandas had learned the feeding schedule as their activity peaked 10–15 min after each event. In contrast, the 10:00 feeding did not produce an anticipatory rise, presumably because it fell within 2 h of the 08:00 feeding and was still within the elevated activity window of the prior feeding. This short two-hour period between feeds likely occurred before the animals could return to a baseline level from which their anticipation could rebuild. This explains why the diel pattern is tri-modal rather than tetra-modal despite four daily feedings. Anticipatory behavior relating to feedings was previously reported for this species [44] and similar influences of keeper actions were also noted by Bugler et al. [43].

To highlight the importance of monitoring wildlife activity patterns as a conservation tool, Bista et al. [45,46], used GPS telemetry, as opposed to motion sensors such as the accelerometers used in this study, to investigate the link between human disturbance and activity/movement patterns of wild *A. f. fulgens*. It was noted that compared with the *A. f. stani* living in protected areas in Sichuan province, the Nepali red pandas living in disturbed areas showed a more uni-modal diurnal pattern. In this group of *A. f. fulgens* the highest activity levels were noted from dawn to 3–4 h after sunrise. The reduction in later-day activities in these red pandas, as well as the associated shift to the uni-modal pattern, was attributed to the anthropogenic pressure of the human-dominated landscape [45,46]. Similar shifts to atypical diel patterns as a result of anthropogenic pressure and habitat disturbances have been noted in numerous species [47,48,49]. In some cases, such as in red deer (*Cervus elaphus*) [50], and wolves (*Canis lupus*) [51], removing the disturbance allowed the animals to revert from an atypical pattern back over time to normal species-specific activity patterns.

As the aim of this present study was to analyze activity patterns of captive red pandas in preparation for future translocations, we theorize that changes in the atypical activity patterns of the two captive individuals, to the more natural pattern seen in wild populations may serve as a positive indicator in terms of how these individuals adjust during the pre-release conditioning period. Furthermore, as part of the post-release monitoring process, determining whether or not, or, how quickly a captive individual transitions into the bi-modal pattern displayed by wild red pandas may therefore be a potential indicator of an individual successfully adapting to the wild habitat. Future research is needed to test this theory. For example, by using triaxial accelerometers on GPS collars, it has been demonstrated that the activity patterns of successfully reintroduced captive giant pandas were more similar to wild giant pandas [11]. In another study, the circadian rhythms of reintroduced captive Milu deer (*Elaphurus davidianus*) were able to revert back to an established natural pattern showing successful reintroduction [17].

In terms of seasonal variation, the three previous studies of wild red pandas in Sichuan province noted clear differences in activity related to seasons, with individuals more active during summer, autumn, and spring [20]. This present study, though limited by the small sample size of one male and one female, noted a sex-biased difference with the female showing a decline in activity (45.94% spring–37.56% winter) related to seasonal change, similar to wild individuals, while the male’s activity (44.81% spring–43.68% winter) was more stable year-round (Table 5). In general, regardless of season, the male individual in this study was more active than the female (Figure 5). Higher activity levels among males were noted by Zhang et al. [20] (49.5% to 47.9%, respectively) but not by Reid et al. [21] (45% to 49%, respectively). Male red pandas typically have a larger home range than females [46,52], this higher activity level in males may be in relation to the need to patrol a larger home range. However, given that red pandas are not sexually dimorphic and previous research based on 17 captive individuals showed no detectable difference in resting metabolic rate between sexes [53], it is possible that differences in activity in this species are related to the individual, not sex; further research is needed to investigate this in more detail.

While the information presented here provides insights for the pre-release management of captive red pandas and has implications for future translocations, there were several limitations. First is the limited sample size, which makes it difficult to draw conclusions based on the activity of only two individuals, a larger sample size would obviously yield more accurate data; however, there are practical limitations on how many individuals of an endangered species can be used for research in a captive setting. Furthermore, the process to train red pandas to allow the placement of GPS collars was labor-intensive and time-consuming, which further limited our sample size and also may not be practical or applicable in most captive settings. Consideration also needs to be made regarding public interpretation of zoo animals wearing GPS collars, especially animals like the red panda that may appear in the illegal pet trade. This was avoided in the present study by working in an off-public area; however, this option may not be available in other institutions. Additionally, due to time constraints and management requirements, we were unable to collect a full year of data; therefore, we were unable to collect activity data for this study during the breeding season.

Another limitation in this study was with the ODBA validation. The video-based behavioral validation (Section 3.2; Figure 3, Table 2) confirms that the four HMM states recover an ordered gradient of activity intensity rather than a noise-driven partition of the ODBA distribution. Furthermore, the validation also reveals two boundaries of this correspondence that should be borne in mind when interpreting the main results. First, behaviorally still observations were more often classified as State 2 (58.9%) than as State 1 (16.0%); State 1 therefore represents ‘absolute minimum ODBA’ rather than a general ‘rest’ category, and inferences should be drawn from the active/inactive binary classification (States 3 + 4 versus States 1 + 2) rather than from State 1 alone. Second, the binary ‘Active’ label captures all non-rest movement rather than locomotion in the strict sense—over 90% of stationary-alert behavior (small head, ear, and tail movements without locomotion) was classified as Active, reflecting the well-established sensitivity of ODBA to small-amplitude movements [40,54]. Direct ODBA-to-energy-expenditure inferences from the activity rates reported here therefore require species-specific calibration that was beyond the scope of this current study. Future studies should consider investigating the links between ODBA, energy expenditure, and environmental covariates.

Despite the above limitations, the present study highlights the potential use of activity patterns derived from triaxial accelerometers in the preparation of captive red pandas for conservation translocations. Taking the activity pattern displayed by the two animals in Panda Valley as an example for pre-release preparation, it is recommended that care should be noted to adjust feeding/staff schedules to more closely match the bimodal activity patterns of wild individuals. As part of the pre-release preparation of red pandas during the reintroduction in India [13], the animals were transitioned from a captive diet to a wild diet over the course of 6 months and then moved to a pre-release site for seven months prior to release into the nature reserve. In the case of the two red pandas presented here, during the diet transition period, the supplemental food should gradually be decreased and removed; however, more bamboo will need to be provided along with the introduction of local fruits and food items. This modified diet should be provided in the morning, while gradually eliminating the 13:30 feed and delaying the 16:30 feed. This change in provisioning schedule may result in a shift from the atypical trimodal pattern to a more natural activity pattern. This pattern can then be monitored as the animal’s transition into the pre-release site as a potential indicator of suitability for release. Post-release, as the animals enter the reserve, resumption of a natural pattern may indicate successful adaptation to the release area. Similar recommendations of adjusting feeding times were made by Bugler et al. [43] in regard to improving the welfare of captive red pandas and are also supported by these current findings.

## 5. Conclusions

Continuous accelerometer-based monitoring of two captive red pandas living in a naturally forested enclosure over ten months yielded an empirically validated four-state activity-intensity classification and a high-resolution diurnal activity profile. This activity profile was dominated by feeding-time entrainment rather than a natural bi-modal pattern observed in wild red pandas. Though based on only two individuals and therefore not generalizable to the subspecies as a whole, this present study provides a validated methodological framework that can be applied at larger scales and a concrete operational signal—the persistence or decay of feeding-entrained peaks—that may serve as a pre-release indicator for future translocation candidates that warrants confirmation in larger samples. The disappearance of the tri-modal pattern post-release, in this view, may therefore track an animal’s behavioral transition from a captive to a free-ranging activity regime.

## Figures and Tables

**Table 1:** Model selection results for Hidden Markov Models with different numbers of activity-intensity states. The four-state model showed the lowest AIC and BIC and was selected.

States	logLik	k	AIC	BIC	ΔAIC
1	113,942.4	2	−227,880.8	−227,862.0	108,833.93
2	113,944.1	7	−227,874.3	−227,808.7	108,840.42
3	113,944.1	14	−227,860.3	−227,729.2	108,854.42
4	168,380.4	23	−336,714.7	−336,499.3	0.00
5	163,343.3	34	−326,618.6	−326,300.2	10,096.07

**Table 2:** Cross-classification of observed behavioral intensity layer (rows) and Hidden Markov Model state (columns), based on *n* = 1441 fifteen-second observations. Cell entries are observation counts. The association was significant (Monte Carlo independence test, B = 10,000; bias-corrected Cramér’s V = 0.599); agreement statistics and the minute-level robustness analysis are reported in Section 3.2. Finer-grained (8 × 4) and active/inactive cross-classifications are reported in Tables S2 and S3.

Observed Intensity\HMM State	State 1	State 2	State 3	State 4	Row Total
Still (r)	28	103	41	3	175
Low (sa)	0	28	214	94	336
Medium (g, scr, e, w)	0	4	302	72	378
High (f, c)	0	0	23	529	552
Column total	28	135	580	698	1441

**Table 3:** Predicted activity probability for the male and the female red panda from the beta-binomial generalized linear mixed model (response: hourly active proportion; random intercept by date). Estimates are mean predicted activity rates with asymmetric 95% confidence intervals back-transformed from the logit scale. Cohen’s h between the two individuals = 0.059, below the conventional threshold for a small effect (|h| = 0.2). With one male and one female, sex and individual identity are confounded; this contrast describes the two animals studied and is not interpreted as a population-level sex effect.

Individual	Activity (%)	95% CI Lower	95% CI Upper
Male	43.78	42.87	44.70
Female	40.85	39.95	41.76

**Table 4:** Beta-binomial generalized linear mixed model of the contrast between the two individuals across seasons. Model: cbind(active, inactive) ~ sex × season + (1|date), family = betabinomial. Estimates are on the logit scale; OR is exp(estimate). Reference levels: sex = Male, season = Spring. As *n* = 1 per sex, these terms describe the contrast between the two individuals rather than a population-level sex effect (see Section 3.5).

Term	Estimate	SE	z	*p*	OR
(Intercept)	−0.208	0.033	−6.24	<0.001	—
sexFemale	0.046	0.047	0.98	0.327	1.047
seasonSummer-Autumn	−0.074	0.045	−1.65	0.100	0.928
seasonWinter	−0.046	0.048	−0.95	0.344	0.955
sexFemale × Summer-Autumn	−0.213	0.064	−3.35	<0.001	0.808
sexFemale × Winter	−0.300	0.068	−4.41	<0.001	0.741

**Table 5:** Seasonal activity rates of the two red pandas studied. Values are model-estimated activity probabilities with asymmetric 95% confidence intervals from the beta-binomial GLMM (Table 4). Within-season effect sizes (Cohen’s h) are reported in Section 3.5.

Season	Individual	Activity (%)	95% CI Lower	95% CI Upper
Spring	Male	44.81	43.20	46.43
Spring	Female	45.94	44.36	47.54
Summer-Autumn	Male	42.98	41.53	44.45
Summer-Autumn	Female	38.93	37.48	40.40
Winter	Male	43.68	42.01	45.38
Winter	Female	37.56	35.96	39.19

## Data Availability

Data is available upon reasonable request to the authors.

## Supplementary Materials

The following supporting information can be downloaded at: https://www.mdpi.com/article/10.3390/ani16111736/s1, Figure S1: Distribution of active and inactive bout durations for each individual. Figure S2: Event-triggered average activity rate around the four daily feeding times. Figure S3: Quantile-quantile plot of normal pseudo-residuals from the four-state Hidden Markov Model fitted to ODBA. Table S1: Bout duration summary statistics. Table S2: Cross-classification of fine-grained ethogram code and HMM state. Table S3: Cross-classification of ethogram code and the binary active/inactive classification. Table S4: Photoperiod-stratified activity probabilities and pairwise contrasts.
