# Supplementary material for: Baseline Activity Patterns of Two Captive Red Pandas (A. f. styani) to Inform Future Conservation Translocations in Sichuan China
Source: Animals (Basel). 2026 Jun 4;16(11):1736. doi: 10.3390/ani16111736 (PMC13255746; doi:10.3390/ani16111736)
Supplement: Supplementary file 1 [file animals-16-01736-s001.zip › animals-4343097-supplementary.pdf]

Supplementary Materials

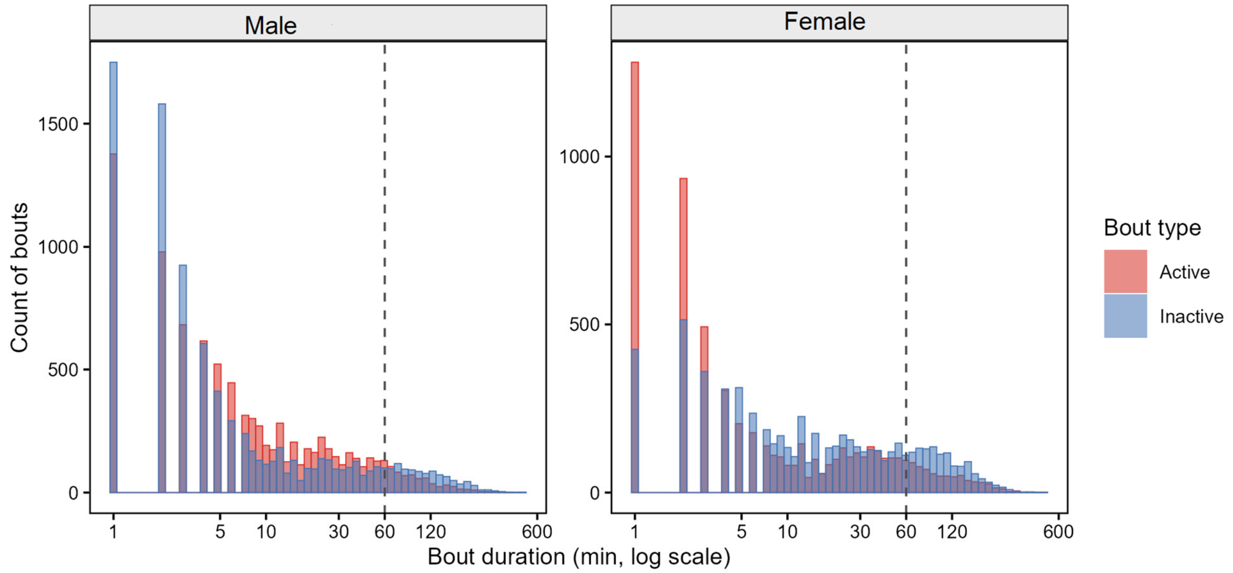

Figure S1. Distribution of active and inactive bout durations for each individual. Bouts are defined as runs of consecutive active or inactive minutes from the binarized state series. The vertical dashed line marks the 60-minute threshold corresponding to the analytical unit (1 hour) used in all generalized linear mixed models. The bulk of the empirical distribution lies well below the 60-minute threshold, supporting the use of the hour as a near-independent analytical unit.

Table S1. Bout duration summary statistics. Values are minutes. Median active bout was 5–6 minutes for both individuals; the 99th percentile of active bouts (151 min for the male, 195.8 min for the female) and inactive bouts (206 min for the male, 211 min for the female) lay above the 60-minute analytical unit but represented < 1% of all bouts.

ID	State	n bouts	Median	P90	P95	P99	Max
Male	Active	9,020	6	49	75.0	151.0	376
Male	Inactive	9,019	4	68	112.1	206.0	477
Female	Active	6,213	5	64	105.0	195.8	406
Female	Inactive	6,212	13	99	138.0	211.0	482

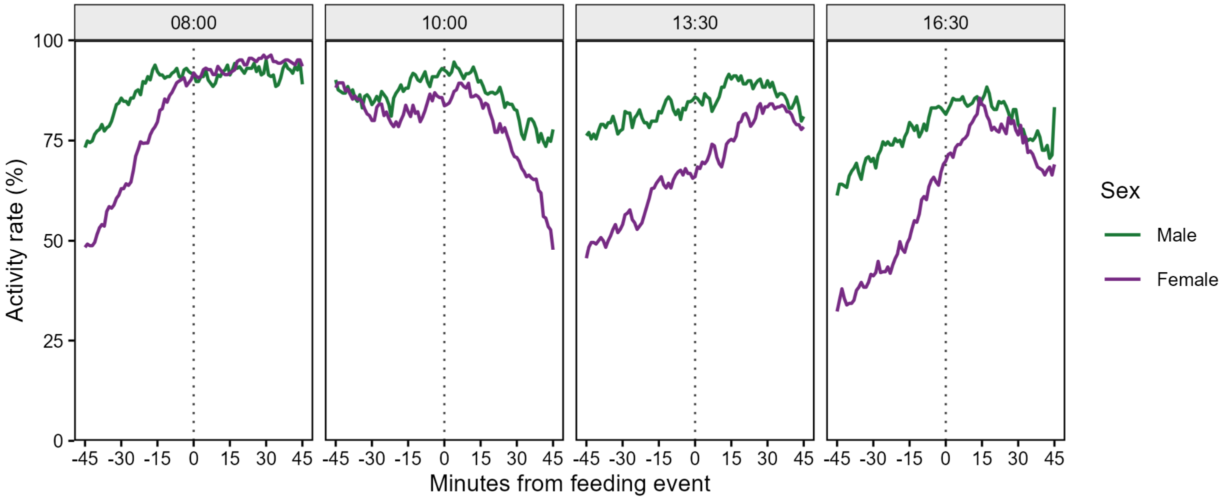

Figure S2. Event-triggered average activity rate around the four daily feeding times. Each panel shows the mean per-minute activity rate by individual in a ±45-minute window centered on the feeding time (vertical dotted line at t = 0). For the female, activity rises from approximately 50% at 45 minutes before feeding to over 85% at the feeding time at the 13:30 and 16:30 feeds; the rise is less pronounced but still visible in the male. The pattern indicates anticipatory behavior and provides correlative support for the interpretation that the tri-modal diurnal pattern reflects entrainment to the feeding schedule.

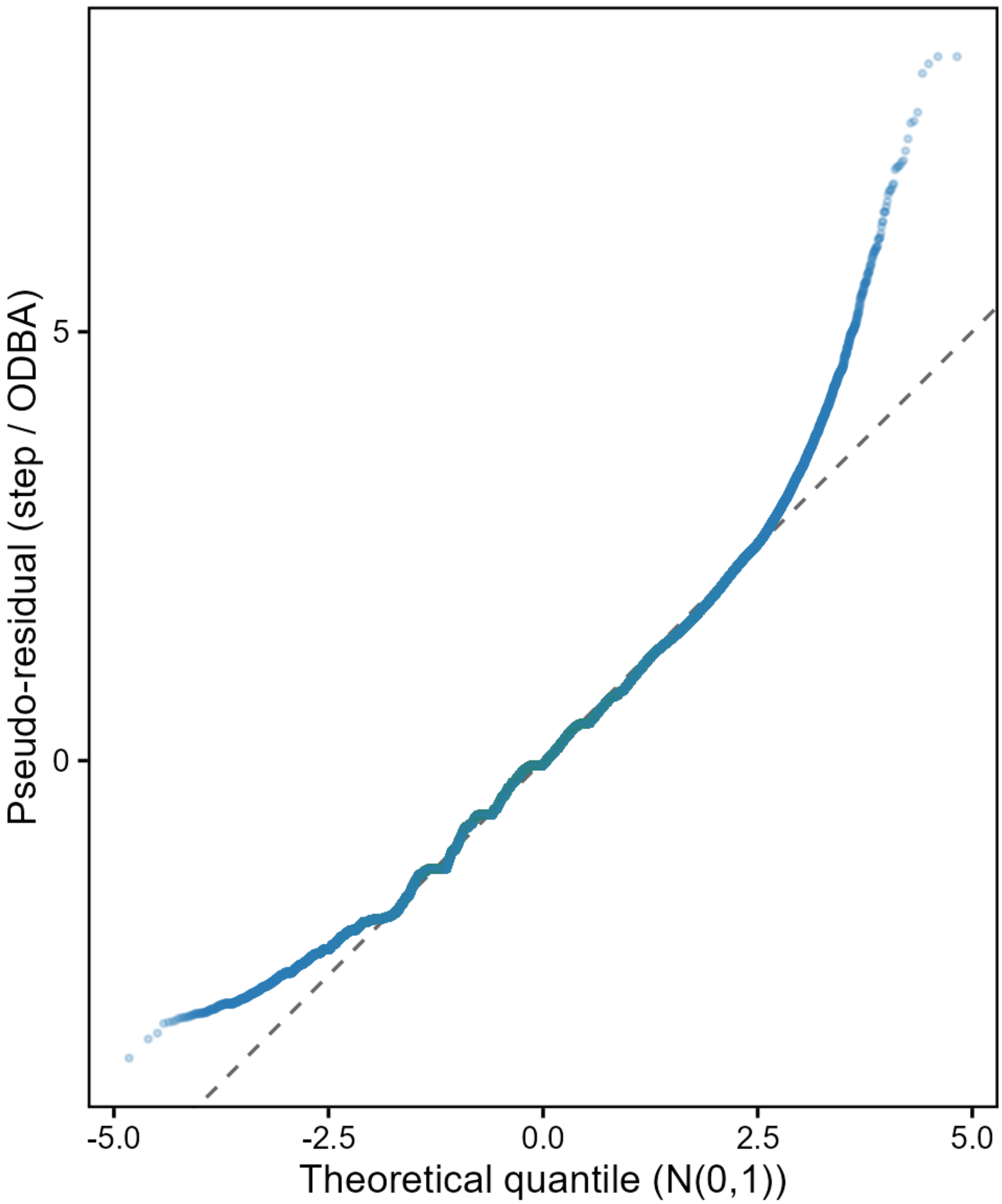

Figure S3. Quantile-quantile plot of normal pseudo-residuals from the four-state Hidden Markov Model fitted to ODBA. Pseudo-residuals are computed by transforming each observation to its corresponding standard-normal quantile under the fitted model; if the model adequately captures the data, pseudo-residuals should follow the standard normal (45-degree dashed line). The bulk of the distribution follows the reference line, indicating an adequate description of the central distribution of ODBA. Some departure from the diagonal in the extreme tails is visible, consistent with the heavy-tailed nature of accelerometer data.

Table S2. Cross-classification of fine-grained ethogram code (rows) and HMM state (columns), based on n = 1441 fifteen-second observations from the validation session. Cell entries are observation counts. Independence test: $chi^2 = 1619.59$, Monte-Carlo $p < 0.001$ (B = 10 000); bias-corrected Cramér's V = 0.609. Behaviour codes: r = rest; sa = stationary alert; g = groom; scr = scratch; e = eat; w = explore (slow walk); f = fast walk; c = climb / branch / jump.

Behavior	State 1	State 2	State 3	State 4	Total
r (rest)	28	103	41	3	175
sa (stat. alert)	0	28	214	94	336
g (groom)	0	3	133	9	145
scr (scratch)	0	1	6	5	12
e (eat)	0	0	95	10	105
w (explore)	0	0	68	48	116
f (fast walk)	0	0	3	471	474
c (climb/jump)	0	0	20	58	78
Column total	28	135	580	698	1441

Table S3. Cross-classification of ethogram code (rows) and the binary active/inactive classification used in the main analysis (Active = HMM States 3 or 4; Inactive = States 1 or 2). Independence test: $chi^2 = 818.41$, Monte-Carlo $p < 0.001$ (B = 10 000); bias-corrected Cramér's V = 0.751. Note that 91.7% of stationary-alert observations are classified as Active, reflecting the sensitivity of ODBA to small head, ear and tail movements (Wilson et al., 2006); the binary "Active" label as used in the main analysis therefore corresponds to "any non-rest movement" rather than to "locomotion" in the strict sense.

Behavior	Inactive	Active	Total	% Active
r (rest)	131	44	175	25.1%
sa (stat. alert)	28	308	336	91.7%
g (groom)	3	142	145	97.9%
scr (scratch)	1	11	12	91.7%
e (eat)	0	105	105	100.0%
w (explore)	0	116	116	100.0%
f (fast walk)	0	474	474	100.0%
c (climb/jump)	0	78	78	100.0%

Table S4. Photoperiod-stratified activity probabilities and pairwise contrasts. Estimates and 95% confidence intervals are model-estimated marginal means from a beta-binomial generalized linear mixed model with photoperiod (three levels: diurnal, crepuscular, nocturnal) as a fixed effect and date as a random intercept; the response variable is the within-hour count of active minutes out of valid minutes. Confidence intervals are asymmetric, computed on the logit scale and back-transformed through the inverse-logit link via the emmeans R package. Pairwise contrasts are reported as odds ratios with 95% CIs (Tukey-adjusted) and as Cohen's h, with the latter interpreted following Cohen (1988): $|h| < 0.2$ = negligible, $|h| < 0.5$ = small, $|h| < 0.8$ = medium, $|h| \geq 0.8$ = large.

Panel A. Estimated marginal means by photoperiod.

Photoperiod	Activity (%)	SE (logit)	95% CI	N (hours)
**Diurnal**	63.61	0.00517	62.59 – 64.61	4,074
**Crepuscular**	60.75	0.00774	59.22 – 62.26	1,920
**Nocturnal**	21.71	0.00360	21.01 – 22.42	5,808

Panel B. Pairwise contrasts between photoperiod windows.

Contrast	Odds ratio	OR 95% CI	p (Tukey)	Cohen's h	Interpretation
**Crepuscular / Diurnal**	0.886	0.820 – 0.957	0.004	0.059	negligible
**Nocturnal / Diurnal**	0.159	0.150 – 0.169	< 0.0001	0.877	large
**Nocturnal / Crepuscular**	0.179	0.166 – 0.193	< 0.0001	0.818	large
